# Catastrophic instability criterion for roadway roof and sidewall rock mass under deep-hole roof blasting in Songshan coal mine

**DOI:** 10.1038/s41598-026-36794-7

**Published:** 2026-01-28

**Authors:** Dongming Guo, Jin Chen, Hengkai Wang, Zhonghua Wei, Qingli Gao

**Affiliations:** Shool of Mechanics and Civil Engineering, China University of Mining & Technology (Beijing), Beijing, 100083 China

**Keywords:** Deep-hole blasting, Layered roof, Roadway sidewall, Cusp catastrophe theory, Stability control, Energy science and technology, Engineering, Natural hazards, Solid Earth sciences

## Abstract

To investigate the catastrophic instability mechanisms of roadway roof and sidewall rock masses under deep-hole roof blasting in coal mines, this study establishes potential energy functions and instability criteria models for layered roof and sidewall strata based on cusp catastrophe theory. First, the layered roof is idealized as a simply supported beam. Considering the coupling effects of deep-hole blasting loads, rock mechanical properties, and support resistance, a total potential energy equation for the system is derived, and the cusp catastrophe equation along with sufficient and necessary conditions for instability are obtained. Second, for the roadway sidewall, a tensile-shear coupled sliding failure mechanical model is proposed to analyze the influence of blasting loads on sidewall stability, and a corresponding catastrophe instability criterion is developed. Through theoretical derivation, the critical maximum explosive charge for both the layered roof and sidewall rock masses is formulated, along with a mechanical criterion for instability judgment. Finally, taking the lower roadway of the 2205 working face in Songshan Coal Mine as the engineering background, theoretical calculations indicate that the maximum critical explosive charge for the roadway is 93.3 kg. Field monitoring shows that, within 1 day after blasting, the roof subsidence increased by 5 mm and the convergence of the two sidewalls increased by 11 mm, indicating that the roadway was only slightly affected by roof deep-hole blasting.This work provides a theoretical basis and engineering guidance for the prediction and control of roadway rock instability under deep-hole roof blasting conditions.

## Introduction

Thick and hard roof coal seams account for more than 30% of China’s coal reserves. Due to their high strength, low joint density, and large bedding thickness, such seams are prone to intense periodic weighting during mining, which can easily trigger dynamic disasters such as rockbursts and surrounding rock instability^1–3^. To address these engineering challenges, deep-hole blasting technology has become the most widely used proactive pressure relief method both domestically and internationally, owing to its efficiency in weakening the roof structure and its economic practicality^4–7^.Deep-hole blasting produces various mechanical effects via explosive shock waves. In proximity to the blast source, it forms crushed zones and radial fracture networks, reducing the structural integrity of the roof. Meanwhile, the majority of the explosive energy is expended in breaking the rock mass and propagating blast-induced fractures, with minimal flyrock and noise generation. Consequently, the resulting stress and seismic waves are characterized by high amplitudes, slow decay rates, and long-distance propagation^8–10^.When these explosion-induced stress waves/seismic waves propagate into the roadway, they impose dynamic disturbances superimposed on the pre-existing static loads borne by the surrounding rock, significantly altering the energy evolution pathway and instability mechanism of the rock mass system. Consequently, they become a critical factor contributing to the catastrophic instability of roadway surrounding rocks^11–14^. Given the above, investigating the failure mechanisms and instability criteria of roadway surrounding rock under deep-hole blasting is of substantial theoretical and engineering significance for ensuring safe and efficient coal mine operations. The failure of surrounding rock in coal mine roadways often exhibits catastrophic characteristics and thus constitutes a geometrically nonlinear stability problem. As a principal analytical framework for nonlinear phenomena, catastrophe theory has been widely applied to rock mass stability analyses. However, existing studies predominantly address roadway instability under static loading, and systematic criteria for evaluating roadway stability under the dynamic loading induced by deep-hole blasting remain insufficient.

Pan et al.^15^ based on the mechanical model of the key stratum zone in roadway surrounding rock, proposed a mechanical prediction model for coal mine rockburst by integrating catastrophe theory. Chen et al.^16^ based on a catastrophe model for slab-buckling impact ground pressure of the surrounding rock mass, derived the expressions of the control variables for both a single rock slab and a composite rock slab under quasi-static loading. Yuan et al.^17^ in order to investigate the safety risk associated with coal uncovering in gas roadways, derived a calculation formula for the critical safety factor of rock pillar thickness based on the principle of energy conservation and catastrophe theory Xu et al.^18^ focusing on impact ground pressure accidents in drill-and-blast roadways, analyzed the evolutionary characteristics of the displacement field, plastic zone, and stress field throughout the entire process of “excavation-deformation-failure-instability-disaster” of deep roadway surrounding rock, and investigated the catastrophe-induced instability mechanism of the roadway.

Zhang et al.^19^ employed cusp catastrophe theory to construct a state recognition model for the irregular coal pillar-roof structure occurring between re-mined roadways and neighboring coal pillars. Based on this model, the stability of irregular coal pillars was classified into different levels. A coupled zoning control technique was proposed and implemented in the mining face. Field monitoring results confirmed the effectiveness of the zoning control method, providing valuable guidance for similar mining practices. Zhao et al.^20^ addressing the challenge of high dynamic pressure and large deformation in gob-side entries under thick coal seam and wide coal pillar mining conditions, employed cusp catastrophe theory to determine the optimal coal pillar width. Theoretical calculations and numerical simulations demonstrated that static blasting for roof cutting can effectively enhance the stability of narrow coal pillars. Wang et al.^21^ based on instability theory and cusp catastrophe theory, determined the stability of mine pillars under asymmetric mining conditions, and identified that the primary factors influencing coal pillar rockburst are the geometric parameters and mechanical properties of the roof–pillar system.

Cao et al.^22^ employed a fold catastrophe model to investigate the mechanism of impact ground pressure induced by strong roof weighting in working faces with thick and hard roofs. The results showed that during the roof weighting process, the fractured coal plate reaches peak stress under impact loading and subsequently enters a post-peak strain-softening stage. When the released elastic strain energy exceeds the energy required for quasi-static failure of the fractured coal plate, the excess energy is rapidly transformed into kinetic energy, resulting in dynamic instability. Yuan et al.^23^ utilized the plastic radius function as the potential function within a cusp catastrophe framework to derive the instability discrimination formula for roadway surrounding rock. Based on this, a catastrophe criterion for deep roadway instability under dynamic disturbance was formulated. Findings reveal that, under extremely high deviatoric stress, the plastic zone in the surrounding rock can rapidly expand in a destructive manner, resulting in significant deformation or sudden failure. Jixun et al.^24^ employed catastrophe theory to introduce a strain energy-based instability criterion, addressing the shortcomings of conventional displacement and plastic zone-based methods in quantitatively characterizing the instability evolution of localized rock mass systems. Xue et al.^25^ investigated the mechanism of coal pillar rockbursts in gob-side entry retaining based on mathematical catastrophe models, and proposed that when the stiffness coefficient of the coal pillar is less than 1 and the external load reaches the peak stress of the coal body, a rockburst is likely to occur. Qin et al.^26^ simplified the roof as an elastic beam and the coal pillar as a strain-softening medium. Using catastrophe theory, they investigated the impact-induced instability of coal pillars, achieving favorable application results in Mentougou Coal Mine.

Previous research has largely focused on the catastrophic instability mechanisms of roadway surrounding rock under static loading. In contrast, limited attention has been given to its stability under deep-hole roof blasting, and criteria for catastrophe instability that account for the combined influence of blasting and support are still lacking.

Songshan Coal Mine, located in Henan Province, China, primarily extracts No. 2–1 coal seam of the Shanxi Formation. The main roof of the roadway consists of thickly bedded fine-grained sandstone. During coal seam extraction, extremely strong mining-induced stress is generated. To sever the mechanical connection between portions of the roadway roof and the goaf roof, deep-hole roof blasting must be carried out at a certain distance ahead of the working face to reduce the influence of mining-induced stress on the roadway.However, the immediate roof strata contain abundant muscovite fragments, lithic particles, and pyrophyllite-like minerals, resulting in weak cementation and a typical layered structure. The coal seam itself is soft and loose, exhibiting low bearing capacity. To prevent catastrophic instability of the weak layered roof and soft coal sidewall of the Songshan Mine roadway under deep-hole roof blasting, this study develops a layered roof surrounding rock mechanical model and a tensile-shear slip instability model for the sidewall based on cusp catastrophe theory. Catastrophe instability criteria for the layered roof and sidewall under deep-hole blasting are formulated and applied in engineering practice, offering a theoretical foundation for analyzing and controlling surrounding rock instability in roadways with weakly layered roofs and soft coal seams.

## Catastrophic mechanical model analysis of layered roof under deep-hole blasting load

### Mechanical model

**Fig. 1:**
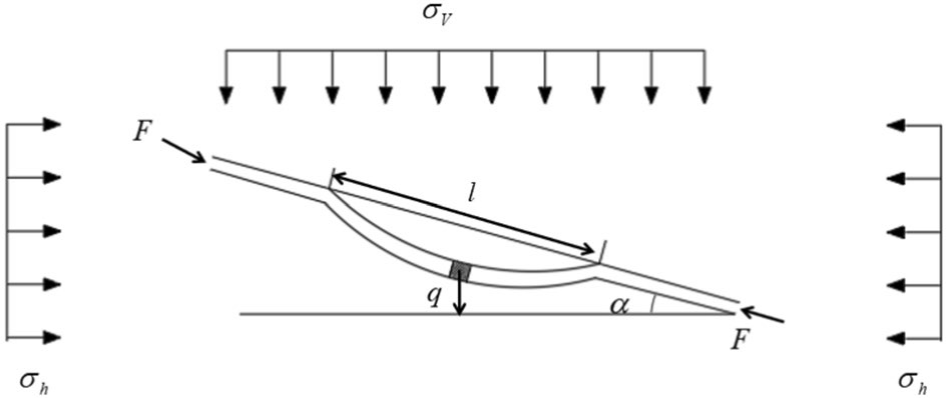
Mechanical model of the stratified roof.

During deep-hole blasting construction in stratified roof rock masses, the combined effects of blasting-induced stress waves and seismic waves can readily induce flexural bulging failure within the layered strata, potentially resulting in roadway instability. Given that the length and width of each rock layer in the stratified roof are significantly greater than their thickness, the deformation and failure behavior can be simplified as a stability problem of a simply supported beam. The flexural deformation reaches its maximum at the mid-span and gradually diminishes toward both ends. Following Reference^27^, the simplified mechanical model of the layered surrounding rock is illustrated in Fig. 1. In this figure: $l$ denotes the rock beam length (m); $h_{{\mathrm{r}}}$ is the thickness of the roof rock beam (m); $b$ is the width (m), taken as 1; $\alpha$ is the inclination angle of the rock layer (°); $E$ is the elastic modulus (GPa); $\sigma_{V}$ and $\sigma_{h}$ represent the vertical and horizontal in-situ stresses (MPa), respectively; $q$ is the distributed load intensity (MPa) arising from the overlying strata and the self-weight of the rock beam; $P_{ir}$ is the upward support stress on the layered roof (MPa); and $F$ is the lateral force acting on the rock beam (kN).

1 $$F = \left[ {q\sin \alpha - \left( {c + q\cos \alpha \tan \phi } \right)} \right]l$$

where:

$c$ is the cohesion of the rock mass (MPa);

$\phi$ is the internal friction angle of the rock mass (°).

Based on the simplified mechanical model of the layered roof surrounding rock, the bending disturbance curve of the rock beam can be expressed as:

2 $$y = \frac{{u_{r} }}{2}\left( {1 - \cos \frac{2\pi x}{l}} \right)$$

In the equation, $u_{r}$ represents the deflection of the layered roof at position $x = l/2$.

### Effects of deep-hole blasting

Blasting Vibration Effect: For calculation simplicity, the vibration generated by blasting on the layered roof is equivalently considered as a static load. The empirical formula is expressed as follows^28^:

3 $$F_{Br} = 2\pi M_{r} \cdot f_{r} \cdot \beta_{Fr} \cdot K_{r} \left( {\frac{{Q^{1/3} }}{{R_{r} }}} \right)^{{\zeta_{r} }}$$

where:

$F_{Br}$ is the blasting equivalent static force acting on the roof beam (N);

$M_{r}$ is the mass of the rock beam (kg);

$f_{r}$ is the dominant frequency of the roof (Hz);

$\beta_{Fr}$ is the equivalent static force coefficient, usually in the range of 0.2–0.3, with smaller values used when structural planes are well developed;

$Q$ is the maximum explosive charge per blast cycle (kg);

$R_{r}$ is the distance from the blast source to the roof, (m);

$K_{r}$ and $\zeta_{r}$ are geological coefficients related to vibration attenuation.

The parameters $f_{r}$, $K_{r}$, and $\zeta_{r}$ are determined through field vibration measurements and curve fitting, which confirms the accuracy and engineering applicability of the above equation.

### Potential energy equation of layered surrounding rock

Based on the mechanical model presented in Sect. 1.1, the total potential energy of the layered roof surrounding rock system is composed of the following parts:

1. Elastic strain energy $V_{1}$ stored from the flexural deformation of the rock beam:
  4 $$V_{1} = \frac{1}{2}\int_{0}^{l} {M_{(x)} } d_{\theta }$$
  where:

$M_{(x)}$ denotes the bending moment at a distance $x$ from the rock beam’s end.

$d_{\theta }$ represents the curvature of the rock beam. Their expressions are given as:

5 $$d_{\theta } = \frac{{M_{(x)} }}{EI}d_{s} \;M_{(x)} = EIy^{\prime \prime }$$

where:

$I$ is the moment of inertia of the rock beam,$I = bh^{3} /12$; $d_{s} = \sqrt {1 + (y^{,} )} d_{x} \approx 1 + 0.5(y^{,} )^{2}$.

Substituting Eqs. (1) and (2) into Eq. (4) yields:

6 $$\begin{gathered} V_{1} = \frac{1}{2}\int_{0}^{l} {EI(y^{,,} )^{2} } d_{s} = \frac{1}{2}\int_{0}^{l} {EI(y^{,,} )^{2} } \sqrt {1 + (y^{,} )^{2} } d_{x} \hfill \\ = \frac{EIl}{8}\left( {\frac{\pi }{l}} \right)^{6} u_{r}^{4} + EIl\left( {\frac{\pi }{l}} \right)^{4} u_{r}^{2} \hfill \\ \end{gathered}$$

where:

$E$ is the elastic modulus of the surrounding rock( GPa).

2) Work done by the lateral force acting on the rock beam, $V_{2}$:
  7 $$V_{2} = \frac{1}{2}F\int_{0}^{l} {(y^{,} )^{2} } d_{x} = \frac{Fl}{4}\left( {\frac{\pi }{l}} \right)^{2} u_{r}^{2}$$

3) Work done by the overlying rock mass, $V_{3}$:
  8 $$\begin{gathered} V_{3} = \frac{1}{2}\int_{0}^{l} {q\left( {l - x} \right)} \left( {y^{,} } \right)^{2} \sin \alpha d_{x} + \int_{0}^{l} {qy\cos \alpha d_{x} } \hfill \\ = \frac{{ql{}^{2}}}{8}\left( {\frac{\pi }{l}} \right)^{2} \sin \alpha \cdot u_{r}^{2} + ql\cos \alpha \cdot u_{r} \hfill \\ \end{gathered}$$

4) Work done by interlayer shear stress of the surrounding rock mass, $V_{4}$:
  9 $$V_{4} = \frac{1}{2}\tau \int_{0}^{l} {\left( {y^{,} } \right)}^{2} dx = \frac{{\tau l^{2} }}{4}\left( {\frac{\pi }{l}} \right)^{2} u_{r}^{2}$$
  where:

$\tau$ denotes the ultimate interlayer shear stress of the rock mass (MPa), determined by the compressive stress between adjacent rock layers and the interlayer friction coefficient. Under blasting disturbances, the laminated surrounding rock can exhibit ultra-low friction, causing notable changes in the ultimate interlayer shear stress. When the layered rock mass becomes relatively loosened, the peak frictional resistance at contact interfaces may decrease drastically, sometimes by severalfold. The ultimate interlayer shear stress of the rock mass can be expressed as:

10 $$\tau = \frac{{n\left( {\sigma_{r} \kappa + c_{r} } \right)}}{2h} \cdot l$$

where:

$\sigma_{r}$ denotes the compressive stress between neighboring rock layers (MPa);

$c_{r}$ is the cohesive strength at the interlayer contact surface (MPa);

$\kappa$ represents the interlayer friction coefficient;

$n$ is the reduction factor of interlayer shear stress caused by blasting.

5) Work done by support resistance $V_{5}$:
  11 $$\begin{gathered} V_{5} = \frac{1}{2}\int_{0}^{l} {P_{{i{\mathrm{r}}}} \left( {l - x} \right)} \left( {y^{,} } \right)^{2} \sin \alpha d_{x} + \int_{0}^{l} {P_{ir} y\cos \alpha d_{x} } \hfill \\ = \frac{{P_{ir} l{}^{2}}}{8}\left( {\frac{\pi }{l}} \right)^{2} \sin \alpha \cdot u_{r}^{2} + P_{ir} l\cos \alpha \cdot u_{r} \hfill \\ \end{gathered}$$

6) Blasting-induced vibrational potential energy $V_{6}$:
  12 $$V_{6} = \frac{1}{2}F_{Br} \int_{0}^{l} {\left( {y{}^{,}} \right)}^{2} d_{x} = \frac{{F_{Br} l}}{4}\left( {\frac{\pi }{l}} \right)^{2} u_{r}^{2}$$

7) The total potential energy equation $V_{r}$ of the layered roof system is:
  13 $$V_{r} = V_{1} - V_{2} - V_{3} + V_{4} + V_{5} - V_{6}$$

Substituting Eqs. (4)-(12) into Eq. (13) gives:

14 $$\begin{gathered} V_{r} = \frac{EIl}{8}\left( {\frac{\pi }{l}} \right)^{6} u_{r}^{4} + \hfill \\ \mathop {}\nolimits^{{\mathop {}\nolimits_{{}} }} \mathop {}\nolimits_{{}} \frac{1}{4}\left[ {4EIl\left( {\frac{\pi }{l}} \right)^{4} - Fl\left( {\frac{\pi }{l}} \right)^{2} - \frac{{\left( {q - P_{ir} } \right)ql^{2} }}{2}\left( {\frac{\pi }{l}} \right)^{2} \sin \alpha } \right. \hfill \\ \mathop {}\nolimits^{{\mathop {}\nolimits_{{}} }} \mathop {}\nolimits_{{}} \tau l^{2} \left( {\frac{\pi }{l}} \right)^{2} \left. { - F_{Br} l\left( {\frac{\pi }{l}} \right)^{2} } \right]u_{r}^{2} - \left( {q - P_{ir} } \right)l\cos \alpha \cdot u_{r} \hfill \\ \end{gathered}$$

### Cusp catastrophe instability model

**Fig. 2:**
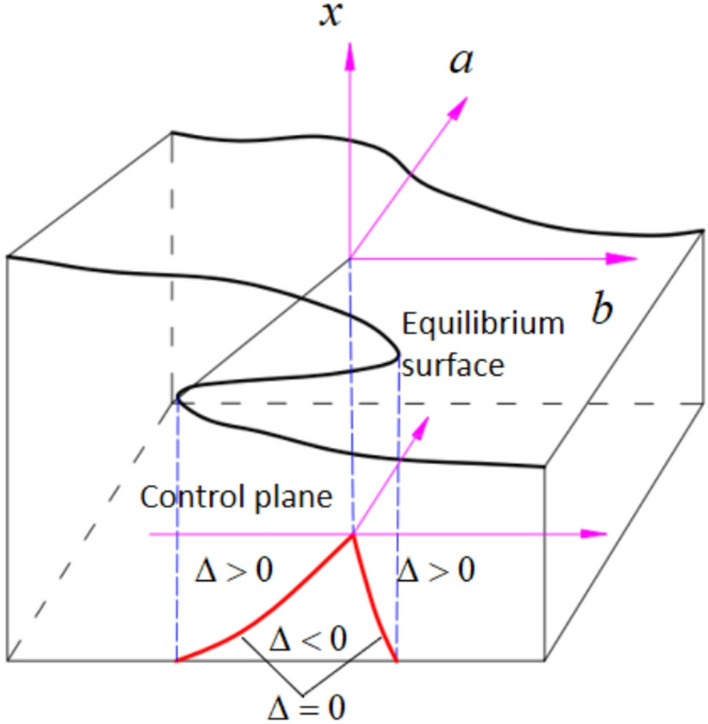
Cusp catastrophe model.

The cusp catastrophe model exhibits high-order characteristics such as hysteresis and divergence. Due to its simple computational structure and ability to abstract instability behaviors, it is one of the most widely applied models in nonlinear stability analysis. This model characterizes the mutation process via a folded equilibrium surface. As illustrated in Fig. 2, the lower layer of the surface denotes the incubation stage of instability in the layered surrounding rock, associated with damage accumulation and degradation, and is regarded as a quasi-stable state. The upper layer reflects the re-stabilized condition following instability, while the middle layer represents the unstable state of the layered surrounding rock.

Based on cusp catastrophe theory, the total potential energy equation of the layered roof surrounding rock system is transformed through variable substitution to obtain its standard cusp catastrophe form. Let:

15 $$x = \left[ {\frac{EIl}{8}\left( {\frac{\pi }{l}} \right)^{6} } \right]^{1/4} u_{r}$$

16 $$\begin{gathered} a = \frac{1}{2}\left[ {4(EIl\left( {\frac{\pi }{l}} \right)^{4} - } \right.Fl\left( {\frac{\pi }{l}} \right)^{2} - \hfill \\ \frac{{\left( {q - P_{ir} } \right)l^{2} }}{2}\left( {\frac{\pi }{l}} \right)^{2} \sin \alpha + \tau l^{2} \left( {\frac{\pi }{l}} \right)^{2} - \hfill \\ \left. { - F_{Br} l\left( {\frac{\pi }{l}} \right)^{2} } \right] \cdot \left[ {\frac{EIl}{2}\left( {\frac{\pi }{l}} \right)^{6} } \right]^{ - 1/2} \hfill \\ \end{gathered}$$

17 $$b = \left( {P_{ir} - q} \right)l\cos \alpha \cdot \left[ {\frac{EIl}{2}\left( {\frac{\pi }{l}} \right)^{6} } \right]^{ - 1/4}$$

The standard form of the cusp catastrophe with $a$ and $b$ as control variables and $x$ as the state variable is expressed as:

18 $$V_{r} = \frac{1}{4}x^{4} + \frac{1}{2}ax^{2} + bx$$

Differentiating Eq. (18) and setting the result to zero gives the equilibrium surface equation containing all equilibrium points, which also reflects the force equilibrium condition:

19 $$V_{r}^{{\prime }} = x^{3} + ax + b = 0$$

The singularity set is given by:

20 $$V_{r} = 3x^{2} + a = 0$$

From Eq. (19), the system’s bifurcation set equation is derived as:

21 $$\Delta = 4a^{3} + 27b^{2} = 0$$

In the equation, $\Delta$ represents the characteristic value of the catastrophe.

### Instability criterion analysis of layered roof strata

As shown in Eq. (21), meeting the bifurcation set equation constitutes the necessary and sufficient condition for catastrophic instability of the roadway. When $\Delta = 0$, the system is at the critical point of instability; for $\Delta < 0$, catastrophic instability occurs; and for $\Delta > 0$, the system stays stable. Hence, the necessary and sufficient mechanical condition for instability of the layered roof surrounding rock can be formulated as:

22 $$\left[ {4EI\left( {\frac{\pi }{l}} \right)^{2} - F - F_{Br} + \tau l - \frac{{\left( {q - P_{ir} } \right)l}}{2}\sin \alpha } \right]^{3} + \frac{27}{2}\left( {P_{ir} - q} \right)^{2} {\mathrm{cos}}^{{2}} \alpha \cdot EI = 0$$

From Eq. (21), it follows that $27b^{2} \ge 0$ always holds in $\Delta$, and $\Delta < 0$ is possible only when $4a^{3} \le 0$. Thus, the necessary condition for instability of the roadway’s layered roof surrounding rock is:

23 $$4EI\left( {\frac{\pi }{l}} \right)^{2} - F - F{}_{Br} - \frac{{\left( {q - P_{ir} } \right)l}}{2}\sin \alpha + \tau l \le 0$$

Based on the preceding analysis, blasting-induced vibrations are regarded as a key external factor influencing the stability of layered roof strata. Consequently, the maximum critical blasting load that the layered roof can bear under certain conditions can serve as a criterion for assessing roof instability. By applying an equivalent transformation to Eq. (22), the maximum critical blasting load $F{}_{Bcr}$ that the layered roof can sustain under specified conditions is obtained as:

24 $$F_{Bcr} = 4EI\left( {\frac{\pi }{l}} \right)^{2} - F + \tau l - \frac{{\left( {q - P_{ir} } \right)l}}{2}\sin \alpha + 3\sqrt[3]{{\frac{{\left( {P_{ir} - q} \right)^{2} }}{4}\cos^{2} \alpha \cdot EI}}$$

From Eqs. (3) and (24), the maximum critical charge quantity $Q{}_{cr}$ that the layered roof can bear under given conditions is expressed as:

25 $$Q{}_{cr} = \left( {\frac{{F_{Bcr} }}{{2\pi f_{r} \beta_{Fr} K_{r} }}} \right)^{{\frac{3}{{\zeta_{r} }}}} R_{r}^{3}$$

From Eqs. (24) and (25), it is evident that the maximum critical charge quantity the roadway’s layered roof can bear at the point of instability is governed by multiple factors. These include the elastic modulus $E$ of the layered surrounding rock, the rock beam length $l$, the beam’s moment of inertia $I$, the blast center distance $R_{r}$ (distance from the charge to the beam), the transverse load $F$ on the beam, the support force $P_{ir}$ on the layered roof, and the rock mass interlayer ultimate shear stress $\tau$. Therefore, the critical charge quantity is variable and cannot be defined by a single parameter. Measures such as increasing $E$ via grouting, reducing roadway span to shorten $l$, enhancing $I$ by anchoring the layered roof with bolts or cables into a composite beam, increasing $R_{r}$ through longer stemming, and improving $P_{ir}$ can effectively strengthen layered roof stability and raise the maximum critical charge quantity $Q{}_{cr}$.

Accordingly, an instability criterion for layered roof strata under the combined effects of blasting load, in-situ stress, and support resistance can be formulated based on the critical charge quantity $Q{}_{cr}$. When $Q > Q{}_{cr}$, the layered roof experiences instability and failure; when $Q = Q{}_{cr}$, it is at the limit equilibrium state; and when $Q < Q{}_{cr}$, the roof remains stable without instability.

In practical deep-hole blasting, to maintain the stability of the layered roof and avoid instability triggered by blasting vibrations—which may cause serious consequences-the maximum critical charge quantity that the roof can sustain is calculated using Eqs. (24) and (25). This serves as a theoretical reference and guidance for determining charge quantities in subsequent deep-hole blasting design.

### Analysis of influencing factors on the stability of layered roof under deep-hole blasting load

According to the analytical conclusions presented in Sect. 1.4, variations in the catastrophe characteristic parameter $\Delta$ can reflect the stability of the layered surrounding rock roof, with a larger value of $\Delta$ indicating better stability of the layered roof strata ^16^. Therefore, the present study will separately investigate the influence of the support resistance $p_{ir}$, explosive charge quantity $Q$, blast center distance $R_{{\mathrm{r}}}$, and elastic modulus $E$ of the stratified roof surrounding rock on the catastrophe characteristic value.

**Fig. 3:**
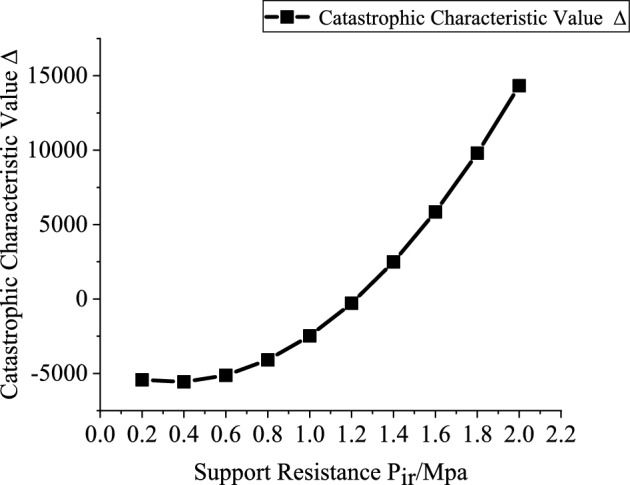
Relationship curve between support resistance $p_{ir}$ and catastrophe characteristic parameter $\Delta$ of the layered roof.

**Fig. 4:**
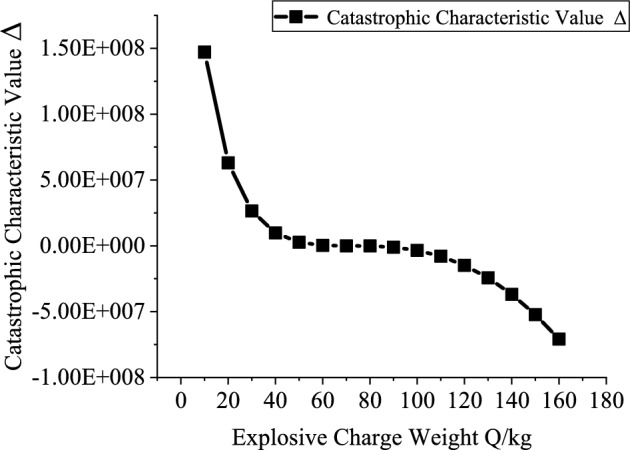
Relationship curve between charge quantity $Q$ and catastrophe characteristic parameter $\Delta$ of the layered roof.

**Fig. 5:**
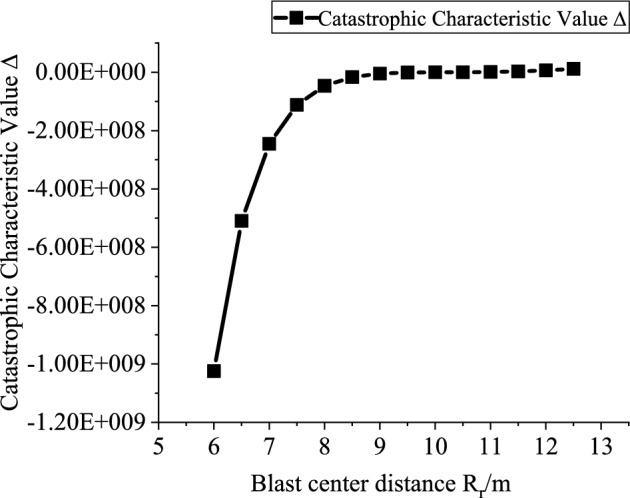
Relationship curve between blast center distance $R_{r}$ and catastrophe characteristic parameter $\Delta$ of the layered roof.

**Fig. 6:**
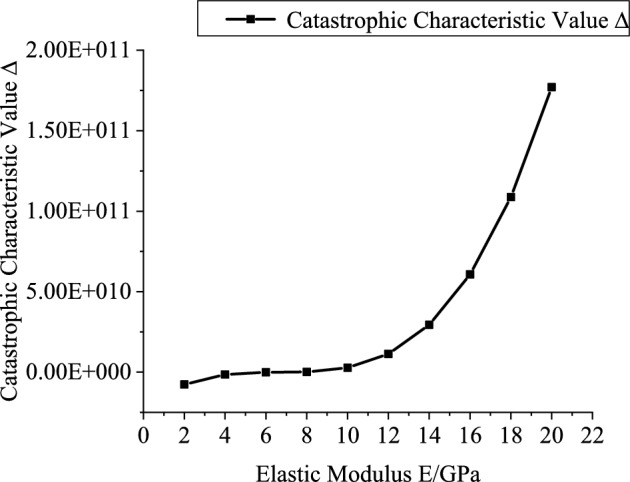
Relationship curve between elastic modulus $E$ and catastrophe characteristic parameter $\Delta$ of the layered roof.

Based on the engineering background, the support resistance $p_{ir}$(0.2–2 MPa, with an interval of 0.2 MPa), explosive charge quantity $Q$(10–160 kg, with an interval of 10 kg), blast center distance $R_{r}$(6–12.5 m, with an interval of 0.5 m), and elastic modulus $E$ (2–20 GPa, with an interval of 2 GPa) are varied to investigate the mutual relationships between each variable and the catastrophe characteristic value. Figures 3, 4, 5 and 6 present the relationship curves for varying $p_{ir}$, $Q$, $R_{{\mathrm{r}}}$, and $E$, respectively. As shown in Figs. 3, 4, 5 and 6 the stability of the stratified roof surrounding rock of the roadway increases with increasing support resistance $p_{ir}$, blast center distance $R_{r}$, and elastic modulus $E$, but decreases with increasing explosive charge quantity $Q$.

All the factors influence the stability of the stratified roof, but their effects on the stability of the system differ in magnitude. A quantitative analysis is therefore conducted based on the sensitivity $S_{{\mathrm{i}}}$

26 $$S_{{\mathrm{i}}} = \left| {\frac{{\Delta K_{i} }}{{K_{i} }}} \right|/\left| {\frac{{\Delta X_{i} }}{{X_{i} }}} \right|$$

where:$\left| {\Delta X_{i} /X{}_{i}} \right|$ is the relative variation rate of each influencing factor, and $\left| {\Delta K_{i} /K{}_{i}} \right|$ is the relative variation rate of the stability-related parameter.

In Figs. 3, 4, 5 and 6, the relationships of support resistance $p_{ir}$, explosive charge quantity $Q$, blast center distance $R_{r}$, and elastic modulus $E$ with the catastrophe characteristic value $\Delta$ of the stratified roof are nonlinear, and inflection points occur in the curvature. Therefore, the sensitivity of each factor should be ranked by segments.

**Fig. 7:**
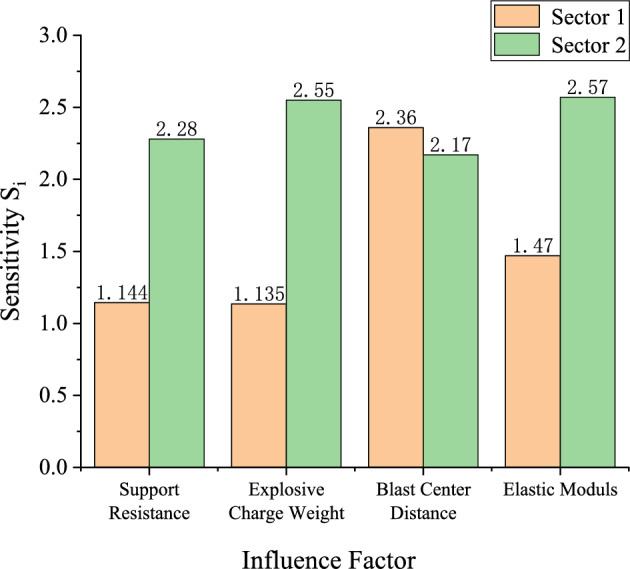
Sensitivity analysis of stability-influencing factors for the stratified roof.

For Segment 1, when $p_{ir}$ < 1.0 MPa,$Q$ < 80 kg,$R_{r}$ < 8.5 m, and $E$ < 14GPa, the sensitivities of the influencing factors calculated from Eq. (26) are shown in Fig. 7. The resulting sensitivity ranking of the stability control parameter of the stratified roof with respect to each factor is: blast center distance $R_{r}$ > elastic modulus $E$ > support resistance $p_{ir}$ > explosive charge quantity $Q$.

Segment 2: when $p_{ir}$ ≥ 1.0 MPa, $Q$ ≥ 80, $R_{r}$ ≥ 8.5 m, and $E$ ≥ 14GPa, the sensitivity ranking of the stability control parameter of the stratified roof with respect to each factor is: elastic modulus $E$ > explosive charge quantity $Q$ > support resistance $p_{ir}$ > blast center distance $R_{r}$. It can be seen that reducing the explosive charge quantity $Q$ and increasing the blast center distance $R_{r}$ are effective in enhancing the stability of the stratified roof.

## Catastrophe mechanical model analysis of tensile-shear slip failure and instability of roadway sidewalls under deep-hole roof blasting load

### Mechanical model

**Fig. 8:**
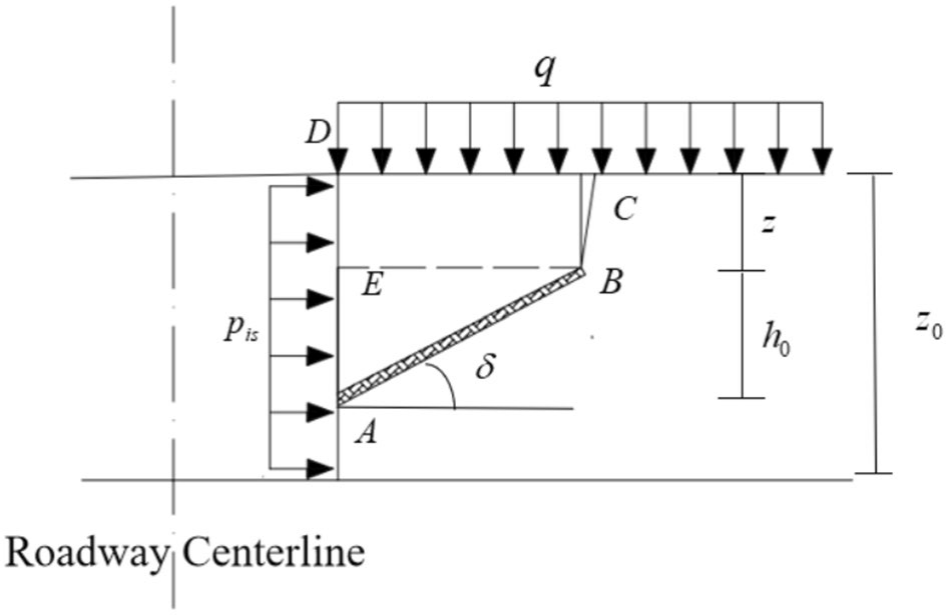
Mechanical Analysis Model of Tensile-Crack and Shear Slip Failure Instability in Roadway Sidewalls.

As blasting dynamic loads are a key factor in causing failure and instability in the sidewalls of deep roadway tunnels, this study establishes a two-dimensional mechanical model for tensile-crack and shear slip failure instability of the sidewalls, as shown in Fig. 8. The following assumptions are made:

1. The failure model of the surrounding rock in the sidewalls of deep roadways under the action of dynamic blasting load on the roof is characterized by tensile-crack and shear coupling sliding.
2. To simplify the calculation, it is assumed that the sliding failure surface of the roadway sidewall consists of two parts: the lower part is the weakened zone (Segment AB), and the upper part is the tensile crack at the rear edge of the sidewall (Segment BC);
3. The length $l_{s}$ of the weakened zone (Segment AB) remains constant.

**Fig. 9:**
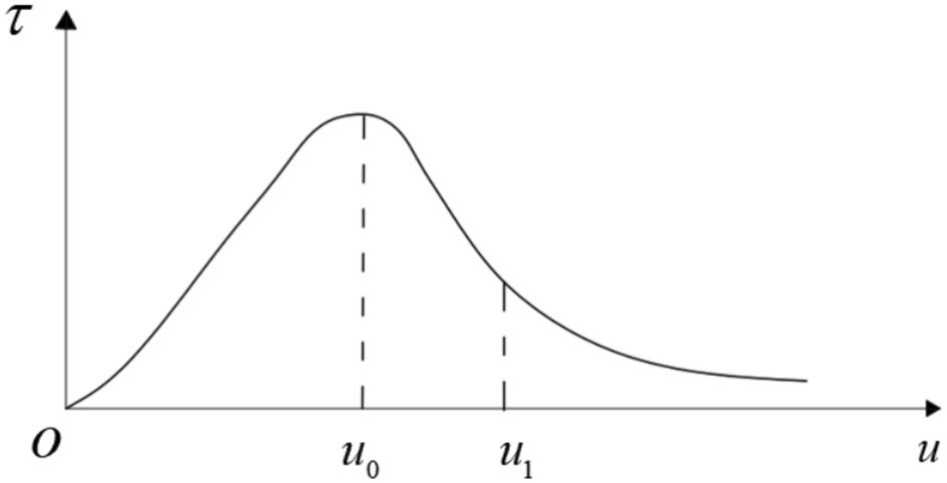
Constitutive Relationship Curve of the Softening Medium Along the Potential Sliding Surface.

As shown in Fig. 8, in the sidewall sliding body ABCD, BCDE represents the tensile crack zone, and ABE represents the shear zone. Here, $q$ is the average load applied to the sliding coal body by the direct roof and overlying strata; the support resistance acting on the roadway sidewall is simplified as a uniformly distributed load $p_{is}$; $W$ is the weight of the sidewall sliding body ABCD; $\gamma$ is the average unit weight of the sliding body; $z_{0}$ is the excavation height of the roadway; $h_{0}$ is the height of the shear slip body front edge; $b_{0}$ is the horizontal collapse width of the sidewall sliding body; $z$ is the depth of the tensile crack at the rear edge of the sliding body; $\delta$ is the angle between the shear slip surface and the horizontal plane, with the thickness of the shear slip surface denoted as $d$; $u_{s}$ is the downward displacement of the sliding body along the sliding trend surface due to its self-weight and external forces. The AB section of the sliding trend surface is the weakened zone, where the medium is more fragmented, the strength is lower, and the impact of blasting vibrations is more intense. It exhibits strain-softening properties, with a friction angle $\phi_{1}$, cohesion $c_{1}$, and length $l_{s}$. The constitutive relationship curve for the AB segment^29^ is shown in Fig. 9.

Based on the geometric relationship in Fig. 8, the weight $W$ of the sidewall sliding body can be expressed as:

27 $$W = \gamma \left( {\frac{{l_{s} h_{0} }}{2} + l_{s} z} \right)\cos \delta$$

The weight $W$ is directed vertically downward. In this study, the sidewall sliding body is assumed to be the quadrilateral ABCD portion shown in Fig. 8.

The constitutive relationship of the medium in the AB segment of the sliding trend surface can be expressed as:

28 $$\tau_{s} = G_{s} \frac{{u_{s} }}{d}e^{{ - u_{s} /u_{0} }}$$

In the equation: $\tau_{s}$ represents the shear stress of the medium in the AB segment; $G_{s}$ is the shear modulus of the medium in the AB segment; $u_{0}$ is the displacement corresponding to the peak point of the constitutive relationship curve of the weakened medium.

Blasting vibration effect. To simplify the calculation, the blasting vibration effect on the sidewall is equivalently treated as a static load, with the empirical formula^28^:

29 $$F_{{Bs}} = 2\pi M_{s} \cdot f_{s} \cdot \beta _{{F_{s} }} \cdot K_{s} \left( {\frac{{Q^{{1/3}} }}{{R_{s} }}} \right)^{{\zeta _{s} }}$$

In the equation:$F_{Bs}$ represents the blasting equivalent static force acting on the roadway sidewall (N);$M_{s}$ is the mass of the rock mass (kg);$f_{s}$ is the primary frequency of the surrounding rock of the roadway sidewall (Hz);$\beta_{Fs}$ is the equivalent static force coefficient of the roadway sidewall, ranging from 0.2 to 0.3, with the smaller value taken when the rock mass has developed structural surfaces;$R_{s}$ is the blast center distance from the roadway sidewall (m);$K_{s}$ and $\zeta_{s}$ are the vibration attenuation geological coefficients of the surrounding rock of the roadway sidewall.Among these, $f_{s}$,$K_{s}$, and $\zeta_{s}$ are obtained through field vibration testing and data fitting.

### Cusp catastrophe model

Through the analysis of the two-dimensional sidewall sliding body dynamic model in Section "Mechanical model", it can be concluded that the total potential energy function of the sidewall system is composed of the following five parts:

1. Strain potential energy $V_{7}$ of the rock mass medium within the weakened zone (Segment AB):
  30 $$V_{7} = l_{s} \int_{0}^{{u_{s} }} {\frac{{G_{s} u_{s} }}{d}} e^{{ - u_{s} /u_{0} }} du_{s}$$

(2) Sliding potential energy $V_{8}$ generated in the direction of displacement $u_{s}$ by the self-weight of the sidewall sliding body and the overlying load:
  31 $$V_{8} = \left( {W\sin \delta + \frac{1}{2}ql_{s} \sin 2\delta } \right)u_{s}$$

(3) Sliding potential energy $V_{9}$ induced by the blasting load in the direction of displacement $u_{s}$:
  32 $$V_{9} = F_{Bs} u_{s}$$

(4) Potential energy $V_{10}$ generated by the support resistance in the direction of displacement $u_{s}$:
  33 $$V_{10} = \frac{1}{2}p_{is} l_{s} \sin 2\delta \cdot u_{s}$$

(5) Potential energy $V_{11}$ dissipated by the sidewall sliding body in overcoming the frictional resistance along Segment AB:
  34 $$V_{11} = \left[ {\left( {W\cos \delta + ql_{s} \cos^{2} \delta + p_{is} l_{s} \sin^{2} \delta } \right)\tan \phi_{1} + c_{1} l_{s} } \right]u_{s}$$

The total potential energy function of the roadway sidewall system is defined as the difference between the strain potential energy of the rock mass medium and the sliding potential energy. Accordingly, the total potential energy function $V_{s}$ can be expressed as:

35 $$\begin{gathered} V_{s} = V_{7} - V_{8} - V_{9} + V_{10} + V_{11} = l_{s} \int_{0}^{{u_{{\mathrm{s}}} }} {\frac{{G_{s} u_{s} }}{d}} e^{{ - u_{s} /u_{0} }} du - \left( {W{\mathrm{sin}}\delta + \frac{1}{2}ql_{s} \sin 2\alpha + F_{Bs} } \right)u_{s} + \hfill \\ \left[ {\frac{1}{2}p_{is} l_{s} \sin 2\delta + \left( {W\cos \delta + ql_{s} \cos^{2} \delta + p_{is} l_{s} \sin^{2} \delta } \right)\tan \phi_{1} + c_{1} l_{s} } \right]u_{s} \hfill \\ \end{gathered}$$

According to the methodology of catastrophe theory, the sliding displacement $u_{s}$ is selected as the state variable. By taking the derivative of the total potential energy function $V_{s}$ in Eq. (35) with respect to $u_{s}$, the equilibrium surface for the stability analysis of the roadway sidewall is obtained as:

36 $$\begin{gathered} V_{s}{\prime} = l_{s} \frac{{G_{s} u_{s} }}{d}e^{{ - u_{s} /u_{0} }} - \left( {W{\mathrm{sin}}\delta + \frac{1}{2}ql_{s} \sin 2\delta + F_{Bs} } \right) + \hfill \\ \left[ {\frac{1}{2}p_{is} l_{s} \sin 2\delta + \left( {W\cos \delta + ql_{s} \cos^{2} \delta + p_{is} l_{s} \sin^{2} \delta } \right)\tan \phi_{1} + c_{1} l_{s} } \right] \hfill \\ \end{gathered}$$

Equation (36) represents the force equilibrium condition. By further taking the third derivative of the total potential energy function $V$, and applying the condition $V^{,,,} = 0$, the following is obtained:

37 $$V_{s}^{{{\prime \prime }}}{^{\prime}} = \frac{{l_{s} G_{s} }}{{du_{0} }}e^{{ - u_{s} /u_{0} }} \left( {\frac{{u_{s} }}{{u_{0} }} - 2} \right) = 0$$

Therefore, the shear displacement at the cusp point of the equilibrium surface is $u_{s} = u_{1} = 2u_{0}$, which corresponds to the inflection point of the constitutive relationship curve of the medium in Segment AB.

Expanding Eq. (36) into a Taylor series with respect to the state variable $u_{1}$ at the cusp point and retaining the first three terms yields the equilibrium surface as:

38 $$\begin{gathered} \frac{2}{3}\frac{{G{}_{s}l_{s} u_{1} }}{d}e^{ - 2} \left\{ {\left( {\frac{{u_{s} - u_{1} }}{{u_{1} }}} \right)^{3} - \frac{3}{2}\left( {\frac{{u_{s} - u_{1} }}{{u_{1} }}} \right) + \frac{3}{2} + } \right. \hfill \\ \frac{3}{2}\frac{{de^{2} }}{{G_{s} l_{s} u_{1} }}\left[ {\frac{1}{2}p_{is} l_{s} \sin 2\delta + \left( {W\cos \delta + ql_{s} \cos^{2} \delta + p_{is} l_{s} \sin^{2} \delta } \right)\tan \phi_{1} + } \right. \hfill \\ \left. {\left. {c_{1} l_{s} - \left( {W\sin \delta + \frac{1}{2}ql_{s} \sin 2\delta + F_{Bs} } \right)} \right]} \right\} = 0 \hfill \\ \end{gathered}$$

By performing a variable substitution in Eq. (38), the equilibrium surface equation can be expressed in the standard form of the cusp catastrophe model:

39 $$V_{s}{\prime} = x^{3} + ax + b$$

40 $$x = \frac{{u_{s} - u_{1} }}{{u_{1} }}$$

41 $$a = - \frac{3}{2}$$

42 $$\begin{gathered} b = \frac{3}{2} + \frac{3}{2}\frac{{de^{2} }}{{G_{s} l_{s} u_{1} }}\left[ {\frac{1}{2}p_{is} l_{s} \sin 2\delta + } \right. \hfill \\ \left( {W\cos \delta + ql_{s} \cos^{2} \delta + p_{is} l_{s} \sin^{2} \delta } \right)\tan \phi_{1} + \hfill \\ \left. {c_{1} l_{s} - \left( {W\sin \delta + \frac{1}{2}ql_{s} \sin 2\delta + F_{Bs} } \right)} \right] \hfill \\ \end{gathered}$$

In the equation:$x$ is the dimensionless state variable; $a$ and $b$ are dimensionless control parameters.

According to Reference^16^, the standard form of the bifurcation set equation in the cusp catastrophe theory model is:

43 $$\Delta = 4a^{3} + 27b^{2} = 0$$

The stability of the roadway sidewall under the combined influence of blasting load and other loads can be analyzed based on the variation of the bifurcation set $\Delta$ and the control variable $a$. When $\Delta < 0$, the system undergoes a catastrophe, and instability-induced sliding occurs in the sidewall. When $\Delta = 0$, the system is in a critical state, and the sidewall is in a marginally sliding condition; in this case, even a slight change in the control variables may lead to a shift in the equilibrium state. When $\Delta > 0$, the system remains stable, and no sliding occurs in the sidewall.

In summary, the condition $\Delta < 0$ represents the necessary and sufficient mechanical criterion for the instability of the roadway sidewall. To satisfy this condition, it is evident that the bifurcation set can only be crossed when $\Delta \le 0$, which corresponds to the control variable $a \le 0$. In this model, the value $a = - \frac{3}{2}$ satisfies the necessary condition for sliding instability of the sidewall under the influence of multiple external loads.

### Analysis of instability criterion for roadway sidewall

From the above analysis, blasting-induced vibrations are identified as a major external factor influencing roadway sidewall stability. Thus, the maximum critical blasting load the sidewall can bear under specific conditions can serve as a criterion for determining its instability. Through an equivalent transformation of Eq. (43), the maximum critical blasting load $F_{{Bc{\mathrm{s}}}}$ that the roadway sidewall can sustain under given conditions is expressed as:

44 $$\begin{gathered} F_{Bcs} = \left( {1 - \frac{\sqrt 2 }{3}} \right)\frac{{G_{s} l_{s} u_{1} }}{{de^{2} }} + \frac{1}{2}p_{is} l_{s} \sin 2\delta + \hfill \\ \left( {W\cos \delta + ql_{s} \cos^{2} \delta + p_{is} l_{s} \sin^{2} \delta } \right)\tan \phi_{1} + \hfill \\ c_{1} l_{s} - \left( {W\sin \delta + \frac{1}{2}ql_{s} \sin 2\delta } \right) \hfill \\ \end{gathered}$$

From Eqs. (27) and (44), the maximum critical charge quantity $Q_{{{\mathrm{c}}s}}$ that the roadway sidewall can bear under specified conditions is given by:

45 $$Q_{cs} = \left( {\frac{{F_{Bcs} }}{{2\pi M_{s} \cdot f_{s} \cdot \beta_{Fs} \cdot K_{s} }}} \right)^{{\frac{3}{{\zeta_{s} }}}} R_{s}^{3}$$

As shown in Eqs. (44) and (45), the maximum critical charge quantity the roadway sidewall can sustain at the onset of instability is affected by various factors, such as the sliding body mass, the overlying load on the sidewall, the depth of rear-edge cracks, the support resistance, and the blast source distance. It is governed by multiple parameters rather than a single one, indicating that the critical charge quantity is variable rather than constant.

Accordingly, an instability criterion for the roadway sidewall under the combined influence of blasting load and other external loads can be formulated based on the critical charge quantity $Q_{cs}$. When $Q > Q_{cs}$, the sidewall experiences instability and failure; when $Q = Q_{cs}$, it is in a marginally stable state (incipient sliding); and when $Q < Q_{cs}$, the sidewall remains stable without instability.

In practical deep-hole blasting, to maintain roadway sidewall stability and avoid tensile-shear slip failure caused by blasting vibrations-which could threaten safety and production-Eqs. (44) and (45) can be applied to determine the maximum critical charge quantity the sidewall can sustain during roof deep-hole blasting. This serves as an important reference for selecting the actual charge quantity in deep-hole blasting design.

### Analysis of influencing factors on the stability of roadway sidewall under deep-hole blasting load

According to the analytical conclusions presented in Sect. 1.4, variations in the catastrophe characteristic parameter can reflect the stability of the roadway sidewall-specifically, a larger value indicates better sidewall stability ^16^.Therefore, this study separately investigates the influence of the sidewall support resistance $p_{is}$, explosive charge quantity $Q$, blast center distance $R_{{\mathrm{s}}}$, internal friction angle $\phi_{1}$ and cohesion $c_{1}$ of the potential sliding surface segment AB on the catastrophe characteristic value $\Delta$.

**Fig. 10:**
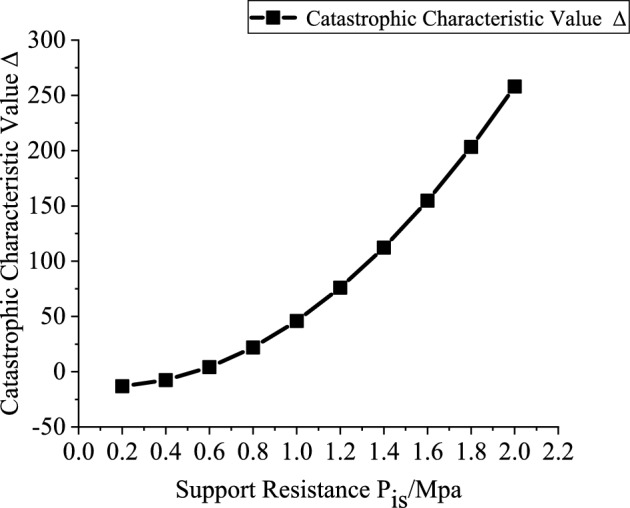
Relationship curve between support resistance $p_{is}$ and catastrophe characteristic parameter $\Delta$ of the sidewall.

**Fig. 11:**
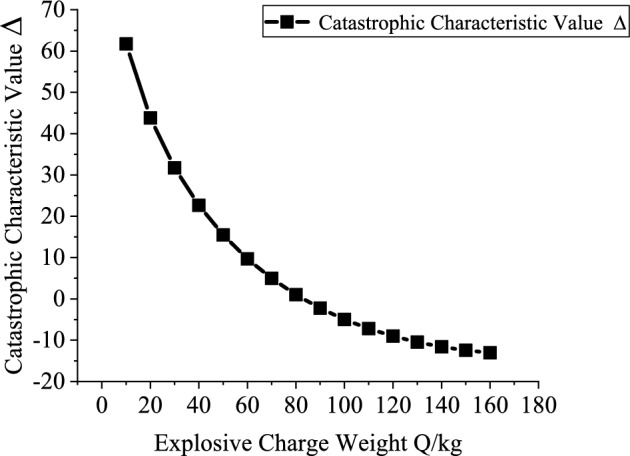
Relationship curve between charge quantity $Q$ and catastrophe characteristic parameter $\Delta$ of the sidewall.

**Fig. 12:**
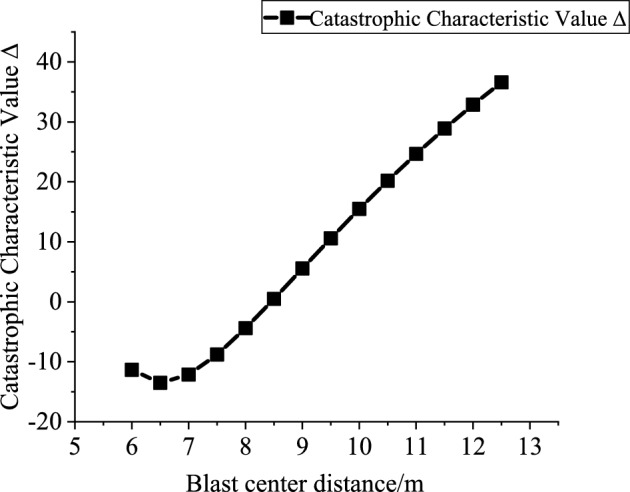
Relationship curve between blast center distance $R_{s}$ and catastrophe characteristic parameter $\Delta$ of the sidewall.

**Fig. 13:**
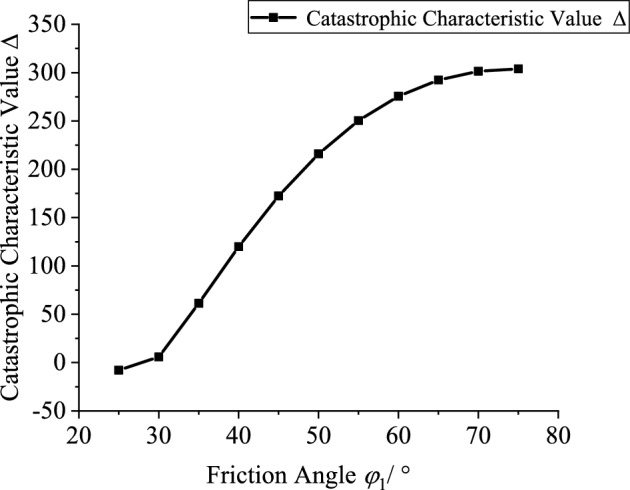
Relationship curve between friction angle $\phi_{1}$ and catastrophe characteristic parameter $\Delta$ of the sidewall.

**Fig. 14:**
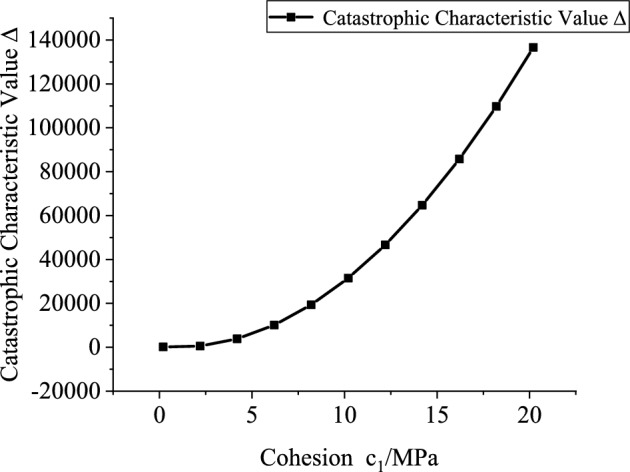
Relationship curve between cohesion $c_{1}$ and catastrophe characteristic parameter $\Delta$ of the sidewall.

Based on the engineering background, the support resistance $p_{is}$(0.2–2 MPa, increment 0.2 MPa), explosive charge quantity $Q$(10–160 kg, increment 10 kg), blast center distance $R_{s}$(6–12.5 m, increment 0.5 m), internal friction angle $\phi_{1}$(25°-75°, increment 5°) and cohesion $c_{1}$(0.2–20.2 MPa, increment 2 MPa) of the potential sliding surface segment AB are varied to investigate the mutual relationships between each variable and the catastrophe characteristic value $\Delta$. Figures 10, 11, 12, 13 and 14 show the relationship curves of $p_{is}$,$R_{s}$,$Q$,$\phi_{1}$,$c_{1}$ with $\Delta$, respectively. As can be seen from Figs. 10, 11, 12, 13 and 14, the stability of the sidewall surrounding rock increases with increasing support resistance $p_{is}$, blast center distance $R_{s}$, internal friction angle $\phi_{1}$ and cohesion $c_{1}$ of the sliding trend surface AB, but decreases with increasing explosive charge quantity $\Delta$.

**Fig. 15:**
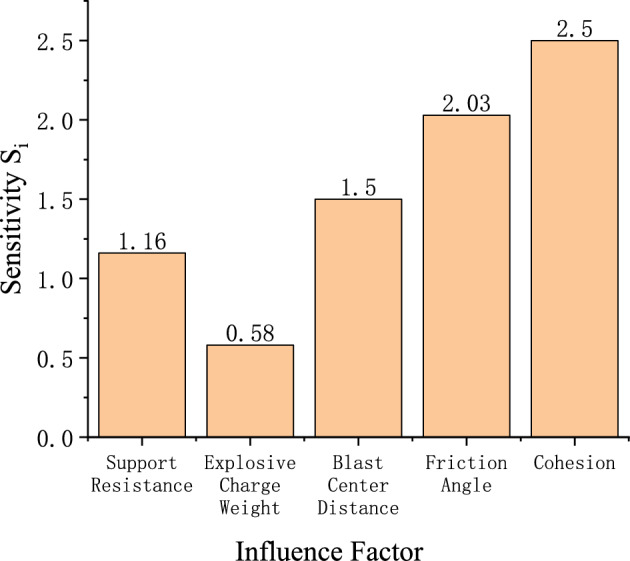
Sensitivity analysis of stability-influencing factors for the roadway sidewall.

All factors influence the stability of the roadway sidewall, but their effects on the system stability differ in magnitude; therefore, a quantitative analysis is conducted based on the sensitivity. The sensitivities of each influencing factor calculated from Eq. (26) are shown in Fig. 15. The sensitivity ranking of the sidewall stability control parameter with respect to each factor is: cohesion $c_{1}$ > internal friction angle $\phi_{1}$ > blast center distance $R_{s}$ > support resistance $p_{is}$ > explosive charge quantity $Q$.It can be seen that the stability of the roadway sidewall is mainly governed by cohesion $c_{1}$, internal friction angle $\phi_{1}$, and blast center distance $R_{s}$. Reducing the explosive charge quantity $Q$, increasing the support resistance $p_{is}$, and enlarging the blast center distance $R_{s}$ are effective measures to enhance the stability of the roadway sidewall.

## Engineering application

### Engineering overview

**Fig. 16:**
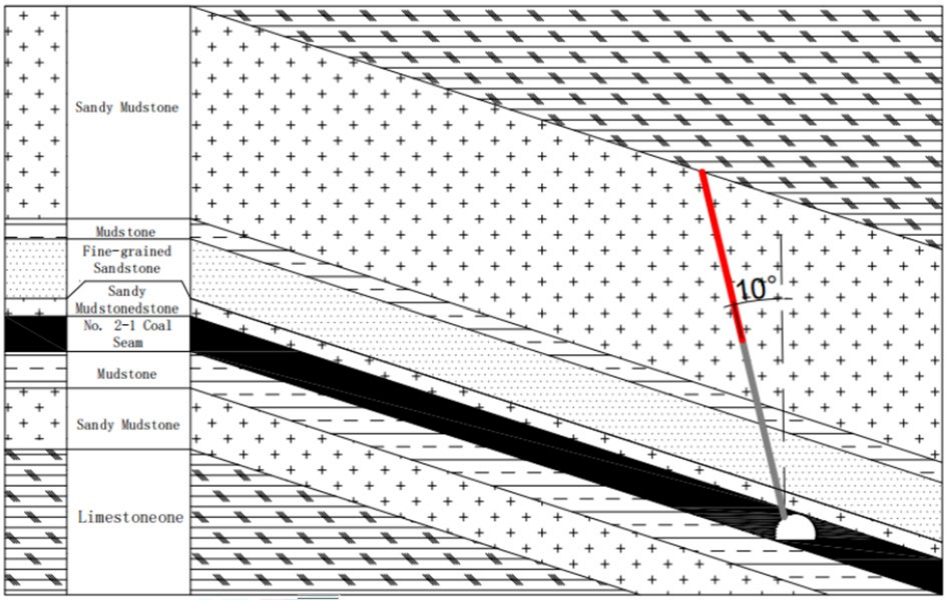
Schematic Diagram of Roof-Cutting Parameters.

**Fig. 17:**
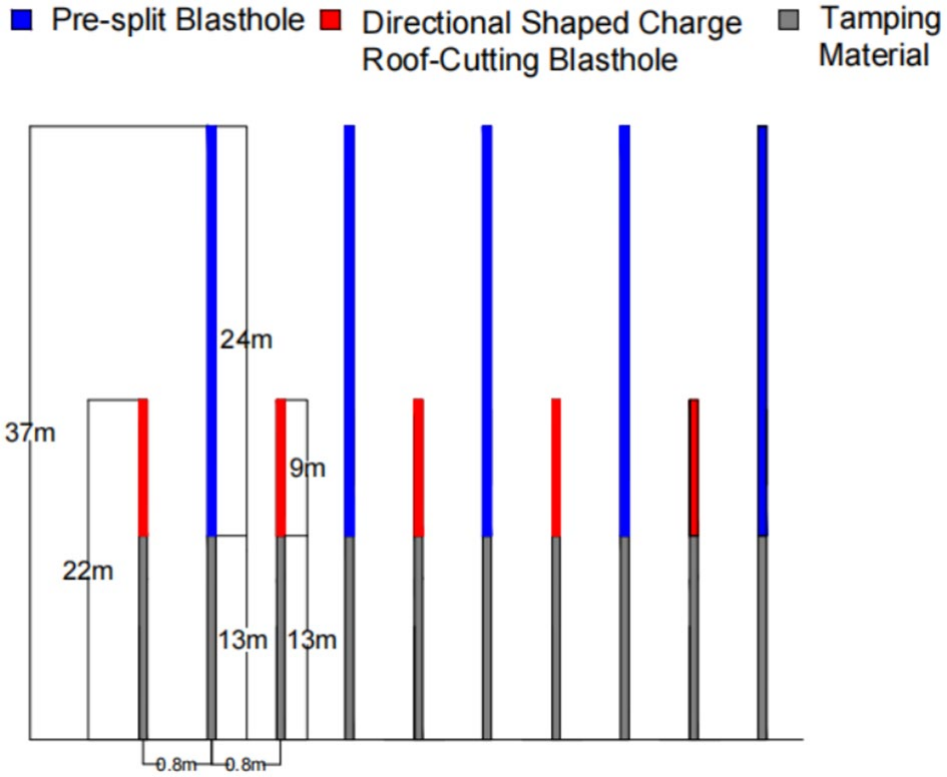
Schematic Diagram of the Pressure Relief Scheme.

Henan Energy’s Songshan Coal Mine lies on the northern limb of the Songshan Anticline and extracts the No. 2–1 coal seam of the Shanxi Formation. The lower roadway of the 2205 working face is arranged along this seam at a burial depth of about 650 m. The seam trends roughly east–west, dips to the north at an average angle of 18°, and has a mean thickness of 4 m. It belongs to the No. 22 mining district of Songshan Mine.The immediate roof contains abundant muscovite fragments, lithic debris, and pyrophyllite-type clay minerals, showing weak cementation and a layered fabric. The main roof consists of thick-bedded, fine-grained sandstone. Strong mining-induced stresses occur during extraction. To break the mechanical linkage between the roadway and goaf roofs and to reduce the influence of these stresses on the roadway, a roof-cutting and pressure-relief method is applied a set distance ahead of the working face. The deep-hole blasting scheme for the roof, which includes the borehole layout and charge structure, is presented in Figs. 16 and 17.

The blasting plan adopts a forward continuous column charge layout, using Class-III coal mine emulsion explosives in cartridges of Φ35 mm × 260 mm. Sympathetic detonation distance ≥ 2 cm; brisance (lead block compression) ≥ 10 mm; detonation velocity ≥ 3200 m/s; work capacity (Trauzl test) ≥ 220 mL; explosive density 1.1 g/cm^3^.Millisecond-delay electronic detonators (segments 1–5) are connected in series. Related blasting parameters are provided in Table 1.Because both the coal seam and immediate roof in Songshan Mine have low strength, only two blast holes per cycle were initiated during early field work to prevent damage to the roof and sidewall during deep-hole blasting, which slowed construction. To ensure safe and efficient application of deep-hole roof blasting in this mine, the lower roadway of the 2205 working face was selected as a case study. Using the instability criteria proposed herein, the stability of the layered roof and sidewall was assessed, and the validity of these criteria was verified against actual roadway field conditions.

**Table 1:** Parameters of the Blasting Pre-split Slotting Test Scheme.

Numbering	Hole spacing (m)	Shaped charge tube(m)	Charge quantity (cartridges)	Explosive Charge Weight(kg)	Stemming Length(m)
Deep Hole	1.6	1.5 × 16	4*16*0.3	19.2	13
Shallow Hole	1.6	1.5 × 6	4*6*0.3	7.2	13

### Field acoustic and vibration testing

In the lower roadway of the 2205 working face, blasting-induced vibrations from roof deep-hole blasting were monitored during field operations to investigate the dynamic response characteristics.Blasting Vibration Testing:As stipulated in the Blasting Safety Regulations, blasting vibration assessment is based on the peak particle velocity (PPV) and the dominant frequency measured at the foundation of the protected structure. Accordingly, vibration monitoring points were installed at both the crown and sidewall of cross-sections located 20–50 m from the working face, spaced at 10 m intervals.

**Fig. 18:**
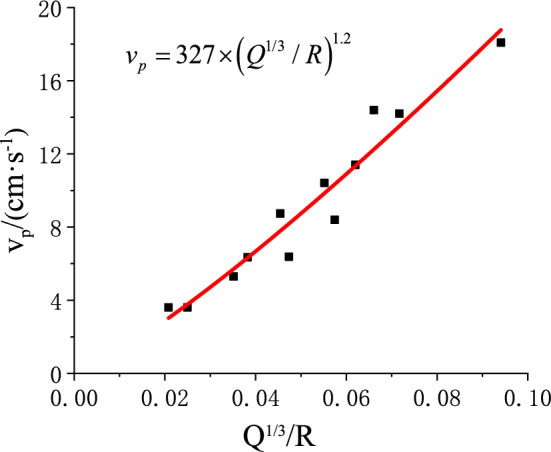
Blasting Vibration Monitoring Data of the Roadway Roof.

**Fig. 19:**
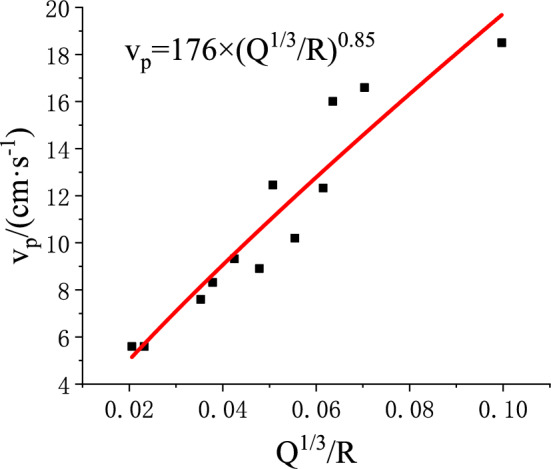
Blasting Vibration Monitoring Data of the Roadway Sidewall.

Three blasting vibration tests were carried out, with monitoring data collected from all designated locations. The peak resultant velocity in three directions was calculated, and curve fitting was applied to obtain the geological attenuation coefficients for use in Eqs. (3) and (29).The results are presented in Figs. 18 and 19, where $v_{p}$ denotes the peak particle velocity induced by blasting, in cm/s.

### Stability analysis

Geological exploration and laboratory testing provided the physical and mechanical parameters of the roof and sidewall in the lower roadway of the 2205 working face, along with the blasting parameters, as listed in Tables 2 and 3. For safety, the blast center distance was set equal to the stemming length.

**Table 2:** Physical and Mechanical Parameters and Blasting Parameters of the Roadway Roof Surrounding Rock.

Physical, mechanical, and stress parameters of the surrounding rock									Blasting and Vibration Parameters			
$E/GP{\mathrm{a}}$	$l/m$	$q/MPa$	$\alpha /(^\circ )$	$\tau /kP{\mathrm{a}}$	$\phi /(^\circ )$	$c/MP{\mathrm{a}}$	$p_{ir} /MP{\mathrm{a}}$	$I/m^{4}$	$K_{r}$	$\zeta_{r}$	$f_{r} /Hz$	$\beta_{Fr}$
2.35	5.57	0.4	18	60	22	0.37	0.25	0.4	327	1.2	25	0.2

**Table 3:** Physical and Mechanical Parameters and Blasting Parameters of the Roadway Sidewall Surrounding Rock.

Physical, mechanical, and stress parameters of the surrounding rock									Blasting and Vibration Parameters			
$W/{\mathrm{kN}}$	$q/MP{\mathrm{a}}$	$p_{is} /MP{\mathrm{a}}$	$\delta /(^\circ )$	$G_{{\mathrm{s}}} /MP{\mathrm{a}}$	$l_{s} /m$	$d/m$	$\phi_{1} /(^\circ )$	$c_{1} /MP{\mathrm{a}}$	$K_{s}$	$\zeta_{s}$	$f_{s} /Hz$	$\beta_{Fs}$
1062	0.4	0.4	18	3000	6	0.02	27	0.016	176	0.85	25	0.2

**Fig. 20:**
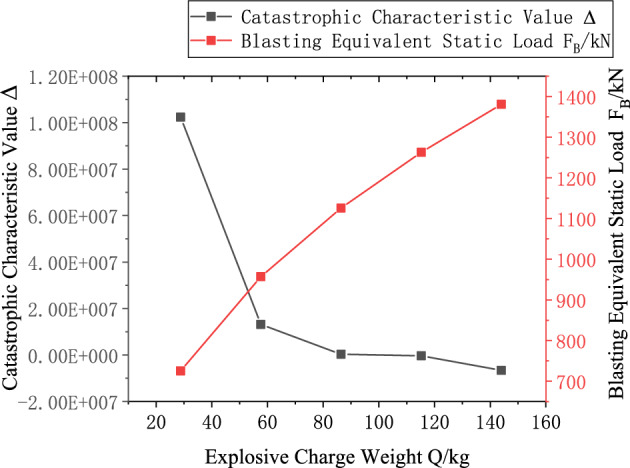
Relationship between Charge Quantity $Q$ and Stability of Layered Roof Surrounding Rock.

**Fig. 21:**
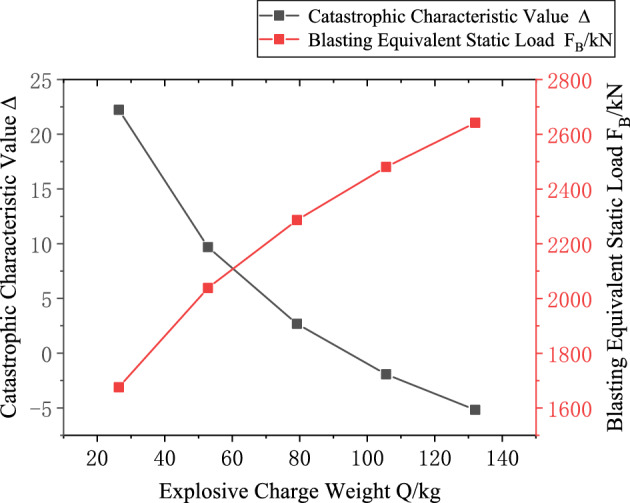
Relationship between Charge Quantity $Q$ and Stability of Sidewall Surrounding Rock.

Using Eqs. (24) and (25), the maximum critical charge quantity for the layered roof was calculated as $Q{}_{cr} = 99.9kg$. From Eqs. (44) and (45), the maximum critical charge quantity for the roadway sidewall was determined as $Q{}_{cs} = 93.3kg$. Substituting the parameters into these equations yielded the catastrophe characteristic parameter $\Delta$ and the equivalent static blasting force $F_{B}$ for the layered roof and roadway sidewall under different explosive charge quantities $Q$, as illustrated in Figs. 20 and 21.

According to the calculation results, in order to prevent sudden failure of the surrounding rock under deep-hole roof blasting, the selection of charge quantity should consider the stability of both the roof and the sidewall. Therefore, the maximum allowable charge should be the smaller of the two respective critical charge quantities. Hence, the maximum critical charge quantity for the roadway is determined to be 93.3 kg.

### Analysis of field conditions

**Fig. 22:**
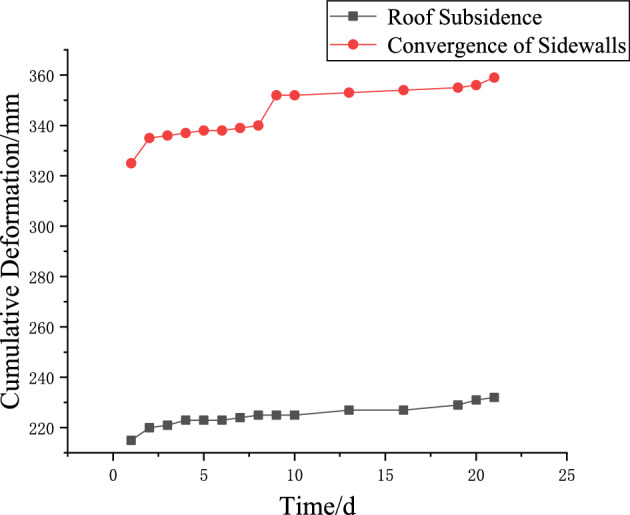
Deformation of Roadway Surrounding Rock.

To achieve safe and efficient coal mine production, and following the above analysis, the initial blasting scheme-two holes per cycle (one deep and one shallow), totaling 26.4 kg of explosives-was revised to six holes per cycle (three deep and three shallow), with a total of 79.2 kg of explosives.To evaluate roadway surrounding rock stability, field monitoring was conducted by measuring roadway deformation. Roof subsidence and sidewall convergence at the monitored section are shown in Fig. 22. Owing to the low strength of the immediate roof and the No. 2–1 Coal Seam at this location, the initial measurements recorded a roof subsidence of 215 mm and sidewall convergence of 325 mm. After deep-hole blasting was applied at this section, subsidence increased by 5 mm and convergence by 11 mm within one day.Over 21 days of monitoring, the deformation rate remained low with only minor additional displacement, indicating that the surrounding rock in this section stayed relatively stable. These results verify that the revised blasting scheme is both safe and effective.

Therefore, the roadway instability criterion under blasting, as derived in this paper based on catastrophe theory, demonstrates a certain level of effectiveness and can accurately predict roadway stability during construction. It is recommended that under similar conditions, stability predictions of the surrounding rock be conducted using a combination of the derived instability criterion and field monitoring. Controlling the explosive charge quantity can effectively reduce vibration effects and improve the safety of roadway excavation.

## Conclusions

1. Using cusp catastrophe theory, a potential energy equation was developed to describe the catastrophic instability of the layered roof surrounding rock under deep-hole blasting. The derived instability criterion for the layered roof strata indicates that blasting primarily affects roof stability through the blasting load, namely, the vibration generated by blasting. As the explosive charge increases, vibration effects intensify, reducing the stability of the layered surrounding rock.
2. A potential energy equation for tensile-shear slip-type failure instability of the roadway sidewall under roof blasting was developed based on cusp theory. The stability of the sidewall surrounding rock increases with greater support resistance and blast center distance, but decreases with increasing charge quantity. Enhancing sidewall stability can be achieved by increasing support resistance, extending stemming length, and reducing the charge quantity per blast.
3. Considering the actual conditions of the lower roadway in the 2205 working face of Songshan Mine, the maximum charge quantity for deep-hole blasting was determined using the proposed catastrophe-based instability criterion. The stability of the roadway roof and sidewall surrounding rock under varying charge quantities was then evaluated. The evaluation results aligned with field observations and monitoring records, confirming the reliability and accuracy of the proposed catastrophe criterion.

## Data Availability

The partial or all data generated or used during the research process can be provided by the corresponding author upon request.
